# Factors affecting registered nurses’ participation and utilisation of clinical research in Namibia

**DOI:** 10.4102/curationis.v48i1.2695

**Published:** 2025-02-25

**Authors:** Malakia K. Mbimbi, Alice Lifalaza, Daniel O. Ashipala

**Affiliations:** 1 Department of General Nursing Sciences, Faculty of Health Sciences and Veterinary Medicine, University of Namibia, Rundu, Namibia

**Keywords:** factors, Namibia, clinical research, hospitals, nurses, evidence-based participation, utilisation

## Abstract

**Background:**

Integrating clinical research into nursing practice is essential for improving patient outcomes; however, various factors can affect nurses’ participation, despite research being one of their key performance areas. In Namibia, little research exists on the factors influencing nurses’ willingness to participate in and use clinical research. The literature indicates that two significant factors affecting registered nurses’ participation in and utilisation of clinical research are insufficient time and a lack of motivation to conduct research.

**Objectives:**

This study explores the factors affecting nurses’ participation and utilisation of clinical research at a regional hospital in Namibia.

**Method:**

The study employed a qualitative, exploratory, descriptive and contextual design to ensure a comprehensive approach. Sixteen participants working at Intermediate Hospital Rundu were purposefully selected. Data were collected via semi-structured interviews and analysed thematically. Interviews were then conducted until data saturation was achieved. Trustworthiness was ensured according to the four principles of Lincoln and Guba. Ethical clearance and permission were granted by the School of Nursing Research Committee. The ethical principles of autonomy, non-maleficence, beneficence, and justice were adhered to.

**Results:**

The study revealed three main themes: individual factors, organisational factors, and research-related factors.

**Conclusion:**

Strengthening individual research capacities, establishing dedicated research infrastructure and resources, and enhancing the communication and dissemination of research findings will foster a research-oriented culture and facilitate the utilisation of clinical research.

**Contribution:**

Understanding these factors will help guide the development of strategies to promote and support nurses’ participation and utilisation of clinical research, enhance evidence-based practice and improve patient outcomes.

## Introduction

Nursing is a dynamic, evidence-based profession that relies on generating and applying new knowledge to improve patient care. It is imperative that nurses continuously participate and utilise research data (Abuhammad et al. 2020; Jabonete & Roxas 2022). Clinical research plays a crucial component of this process; it helps to identify best practices, inform decision-making and drive innovation in healthcare delivery. Wallen and Fisher (2018) define clinical research as a form of clinical nursing practice, with a speciality focus on research implementation and the care of subjects participating in clinical research. Grys (2022) states that integrating clinical research into nursing practice is essential for improving patient outcomes, enhancing the quality of care and advancing the nursing profession. Nursing research involves a structured investigation to understand matters that are significant to nurses, such as nursing practice, nursing education and nursing administration (Polit & Beck 2021).

Nursing research utilisation is essential for integrating, disseminating and applying research findings to substantially impact current nursing practices (Bal & Sahiner 2019). According to Chalmers et al. (2023), healthcare professionals must participate and utilise clinical research data in their quest to improve quality healthcare delivery. Therefore, conducting health science research leads to healthcare improvements, earning and defending a professional status, establishing scientifically defensible reasons for healthcare practices, finding ways to enhance the cost-effectiveness of healthcare services, providing a base for standard-setting and quality assurance, providing evidence of weaknesses and strengths within the field, providing evidence in support of requests for resources (Bonfim et al. 2023) and providing a base for self-correction of misinterpretations and myths (Martin 2022).

In a global context, nurses’ participation in research is hindered by numerous factors, such as insufficient time, organisational policies, mentorship, funding and knowledge (Smith and Johnson, 2023). Evidence-based practice is widely recognised as vital for delivering high-quality patient care (Dagne & Ayalew 2020). The level of research participation and utilisation among nurses has been indicated to vary across different regions and healthcare settings (Oyediran et al. 2021). Several studies show that few nurses participate in and implement research in their practices (Kyei et al. 2023; Poorchangizi et al. 2019; Rojaye & Netangaheni 2023).

In the African context, studies have shown that nurses’ participation and utilisation of research findings are significantly hampered by their limited perception of the advantages of research, understanding of time as a factor for reading scientific articles, lack of support and also beliefs that research is not a part of the nursing role (Kyei et al. 2023; Nkrumah et al. 2018). Additionally, a lack of knowledge about conducting research has also been identified as a barrier to nurses’ participation in clinical research (Vas et al. 2021). Musumadi et al. (2023) add that in the category of educational preparation, nurses lack training and mentorship with research experience and have uncertainty about how to participate in research. For nursing practice to be evidence-based and high-quality, nurses need access to research evidence and the skills to critically appraise and apply it (LoBiondo-Wood & Haber 2021).

In Namibia, research is one of the key performance areas in the job description of registered nurses. It is also a requirement for obtaining an undergraduate bachelor’s degree in nursing (Ashipala & Livingi 2021). In Namibia, research has been highlighted as a major contributing factor to cost reductions, enhanced productivity (Shifaza, Evans & Bradley 2014) and the promotion of high-quality healthcare, ultimately leading to improved patient outcomes (Nor et al. 2021). This underscores the importance of nurses participating in research as part of their clinical practice (Sowunmi et al. 2021). Currently, in Namibia, there is limited information on the factors that affect their participation in and utilisation of clinical research. Moreover, Namibia has no nationwide research funding mechanism, hence research in the country is improved through the development of sustainable national funding structures and mechanisms (Kgabi 2011). This study thus aimed to explore and describe the factors that affect registered nurses’ participation in, and utilisation of, clinical research at Rundu Intermediate Hospital in Namibia.

## Research methods and design

This study used a qualitative, exploratory, descriptive and contextual design which allowed the researchers to explore and describe the factors affecting nurses’ participation in, and utilisation of clinical research at a regional hospital in Namibia. Qualitative research made it possible to examine their subjective human experiences using non-statistical or non-numerical counting methods of analysis (Daneshgar 2023; Roestenburg, Strydom & Fouché 2021; Salmons 2023). This approach enabled the researcher to evaluate individuals’ comprehension of their experiences, perspectives and environment concerning a particular phenomenon (Brink, Van Der Walt & Van Rensburg 2018).

### Setting

The research was carried out at the primary government hospital in the Kavango region, located on the border with Angola. With 600 staff members and approximately 300 beds, this facility is a referral centre for nearby district hospitals and a provider of specialised medical services. Moreover, it is an educational institution where students pursuing nursing, medicine, physiotherapy, occupational therapy, pharmacy and dentistry gain practical experience. This hospital provides diverse healthcare offerings, including primary care, maternal and child health services, HIV and AIDS treatment and prevention, tuberculosis control, mental health services and chronic disease management. The researcher chose this setting because it offers unique characteristics for conducting the research, such as easy access to essential participants needed to answer the research questions, convenient location and the ability to gain permission to conduct the study. Additionally, the setting had a sufficient population to obtain data, and there was no cost involved in meeting the participants for inclusion in the study.

### Sampling

The study targeted the population of registered nurses at the hospital. Sixteen registered nurses (age: 24–42 years old) who met specific inclusion criteria were purposively selected to participate in the study. To be eligible for inclusion in this study, participants had to be working at Intermediate Hospital Rundu for at least one or more years and signed the informed consent. Interviews were then conducted until data saturation was achieved at the 16th participant, as no new themes, insights or information emerged beyond this point.

### Data collection

The data for this study were collected via semi-structured interviews between October and November 2022. The researcher wrote to managers of Rundu Intermediate Hospital for permission to post flyers on available notice boards in staff tea rooms, boardrooms and all around the hospital to invite potential participants. The flyers contained the researcher’s contact details, which willing participants used to reach out to the researcher. Willing participants were invited to an information session to learn about the study’s purpose and to ask any questions they had. An interview guide was developed based on the research question and objectives, and literature review. Three pilot interviews were carried out to assess the guide’s ability to collect pertinent data; however, no changes were needed. The data from the pilot interviews did not become part of the main study. They willingly signed an informed consent form and permitted recording. The interviews were conducted in English as all the participants speak, write and understand the English language. The interviews lasted 30–45 min, with probing questions asked to encourage in-depth responses. The interviews were conducted in a private, comfortable setting at Intermediate Hospital Rundu to ensure confidentiality and encourage open and honest responses (Rojaye & Netangaheni 2023; Sowunmi et al. 2021). The key questions posed were:

- *What factors affect registered nurses’ participation in, and utilisation of, clinical research?*
- *What would encourage registered nurses’ participation in, and utilisation of, clinical research?*

### Data analysis

The data analysis involved transcribing the interviews, manually coding the data and applying reflexive thematic analysis. A reflexive thematic analysis technique, utilising an inductive approach, was employed to examine the diverse range of participant experiences and perspectives of the phenomenon (Braun & Clarke 2019). This process involved immersion in the data, identifying patterns and themes, and returning to the relevant literature to refine and validate the emerging themes. The data analysis adhered to Braun and Clarke’s six-phase thematic analysis approach, that is, becoming familiar with the data, categorising them, deriving themes, reviewing them, defining and naming them, and preparing a comprehensive report. Before data collection, the researcher embraced a self-reflective ‘bracketing’ whereby their own beliefs, preconceived responses and biases regarding the topic were set aside to ensure an open-minded approach. Furthermore, developing a coding tree effectively illustrated the sub-themes’ evolution from highlighted themes, substantiating their organic emergence from the collected data.

### Trustworthiness

The trustworthiness of the study was ensured by using Lincoln and Guba’s (1985) model, which establishes the credibility, dependability, confirmability and transferability of a study (Korstjens & Moser 2017; Brink et al. 2018). Credibility in this study was achieved through prolonged engagement with, and persistent observation of, the participants. The researcher remained in the field for 4–6 weeks in order to gain an in-depth understanding of the phenomenon. A tape recorder was used during the individual interviews, and the tapes were transcribed. The recordings and transcripts were compared to confirm that they were from the same participants. Dependability was achieved through the use of audio recordings and transcripts can be made available upon enquiry. The data collected is also supported with participant quotes, and the data collection methods are comprehensively described. Transferability was achieved through a complete description of the research design and methodology used, as well as through the purposive selection of participants. The data collected were described as accurately as possible; full or thick descriptions of the experiences of the participants can be made available upon enquiry. Therefore, researchers can decide on the transferability of research findings to other contents. Data collected were kept available for audit trails so that participants could relate to the interpretations. In addition, verbatim transcription of the interviews and re-reading of these and the field notes enabled the researcher to get a better understanding of what the participants said about the factors affecting nurses’ participation and utilisation of clinical research at a regional hospital in Namibia.

### Ethical considerations

Ethical approvals were obtained from the University of Namibia (UNAM) Health Research Ethics Review Committee (HREC) Reference Number 137/2022, as well as from the Ministry of Health and Social Services Research Committee (MoHSSRC) Reference 22/4/2/3. Participants signed informed consent before participation. Respect for people was observed throughout the research process by adhering to ethical principles (respect for persons, beneficence and justice) (Kessio & Chang’ach 2020). Adherence to the ethical principle of respect for people was paramount. The researcher obtained informed consent from all the participants, which ensured that they were fully informed of the study’s aim and benefits, and voluntarily agreed to participate in the study. There was no risk for participating in this study as the study did not include tests or human trials. Participants in this study benefited indirectly as the findings will be used to improve the quality of patient care. Additionally, the confidentiality of the participants’ personal information was guaranteed; all audio recordings, interview transcripts and field notes are securely stored in a password-protected computer accessible exclusively to the researcher and the study supervisors. The data will be kept for a period of 5 years in accordance with institutional research policy.

## Results

### Demographic characteristics

Sixteen registered nurses employed at the local public hospital participated in the research. Their ages ranged from 24 years to 42 years old, with an equal split between males and females. The nurses were evenly distributed across various departments and most had a bachelor’s degree in nursing science. Their work experience spanned from 1 to 25 years, and none had undertaken any operational research (Table 1).

**TABLE 1:** Participants’ demographic characteristics.

Code	Age (years)	Race	Gender	Department or ward	Cert., Dip., Degree or Master	Years of experience	No. of research
P1	37	Black	Male 1	Customer care	Diploma in general nursing science and midwifery, post-graduate specialisation in clinical instructions	12	None
P2	24	Black	Female	OPD or casualty	Bachelor’s degree in nursing science clinical honours	2	None
P3	34	Black	Male	Male ward	Bachelor’s degree in nursing science	1	None
P4	24	Black	Female	Post-natal maternity ward	Bachelor’s degree in nursing science clinical honours	1	None
P5	29	Black	Male	TB ward	Bachelor’s degree in nursing science	5	None
P6	24	Black	Female	Maternity post-natal	Bachelor’s degree in nursing science	1	None
P7	33	Black	Male	OPD or casualty	Bachelor’s degree in nursing science	3	None
P8	42	Black	Female	Maternity antenatal	Bachelor’s degree in nursing science	17	None
P9	36	Black	Male	Operating theatre	Bachelor’s degree in nursing science, with a specialisation in Theatre Operation	10	None
P10	38	Black	Female	Surgical ward	Bachelor’s degree in nursing science	14	None
P11	32	Black	Male	TB ward	Bachelor’s degree in nursing science	7	None
P12	35	Black	Female	High care or ICU	Bachelor’s degree in nursing science with a Specialisation Diploma in Intensive Care Unit	11	None
P13	36	Black	Male	Male ward	Bachelor’s degree in nursing science	14	None
P14	41	Black	Female	Paediatric ward	Bachelor’s degree in nursing science	17	None
P15	39	Black	Male	Male ward	Bachelor’s degree in nursing science	15	None
P16	34	Black	Female	Nursing management	Bachelor’s degree in nursing science, with a specialisation in mental health	10	None

Cert., certificate; Dip., diploma; No., number; TB., tuberculosis; ICU, intensive care unit.

### Themes and sub-themes

Three main themes emerged from the data analysis, each with several sub-themes: (1) individual factors; (2) organisational factors and (3) research factors (Table 2).

**TABLE 2:** Themes and sub-themes.

Themes	Sub-themes
1. Individual factors	1.1 Deficiency in research awareness and skills 1.2 Professional experience and role 1.3 Absence of motivation and self-efficacy 1.4 Scarcity of mentors and professional support 1.5 Limited personal benefit
2. Organisational factors	2.1 Inadequate financial and material resources 2.2 Lack of dedicated research time 2.3 Absent research board committee and infrastructure 2.4 Absent leadership and management support 2.5 Sub-optimal research culture 2.6 Lack of in-service training on the clinical research process
3. Research factors	3.1 Complexity of the research process 3.2 Lack of communication and feedback 3.3 Insufficient guidance from research supervisors 3.4 Absence of institutional mechanisms for research dissemination.

#### Theme 1: Individual factors

This theme captured the personal and professional attributes that influenced the nurses’ involvement in research activities. Sub-themes encompassed: (1.1) deficiency in research awareness and skills; (1.2) professional experience and role; (1.3) absence of motivation and self-efficacy; (1.4) scarcity of mentors and professional support; and (1.5) limited personal benefit.

**Sub-theme 1.1:** Deficiency in research awareness and skills

The participants expressed a lack of formal knowledge of research methodologies and limited knowledge about the processes involved in conducting nursing research:

‘The curriculum during my nursing education covered research methods or provided opportunities to develop research skills, however it was just hard to understand.’ (P14, Female, 17 years of experience)

‘I am not aware of the research activities and resources available within our institution.’ (P7, Male, 3 years of experience)

‘Even if you find a problem here, you don’t know if the problem is valuable to be conducted research.’ (P2, Female, 2 years of experience)

**Sub-theme 1.2:** Professional experience and role

Participants expressed less experience and felt less empowered to engage in research activities:

‘I am still trying to find my feet in this profession, so research is not a priority for me at the moment.’ (P2, Female, 2 years of experience)

‘I am primarily focused on learning the basics of nursing practice and do not have the time or confidence to take on research projects.’ (P4, Female, 1 year of experience)

‘Research seems important, but I feel like I lack the skills and knowledge to get involved right now. My focus is more on improving my practical skills.’ (P6, Female, 1 year of experience)

**Sub-theme 1.3:** Absence of motivation and self-efficacy

Participants highlighted a lack of intrinsic motivation to conduct research, coupled with a low sense of self-efficacy in their ability to undertake research projects successfully:

‘I lack the confidence to initiate or participate in research activities, and I do not know where to start or who to approach here at this hospital.’ (P2, Female, 2 years of experience)

‘Personally, I would not want to do research because I’m exhausted from school for now … and too much stress from the workload … And there’s no support.’ (P3, Male, 1 year of experience)

‘Number one is lack of understanding regarding the importance of nursing research at the hospital level from those that are supervising us or from the matron and the other management kinder.’ (P1, Male, 12 years of experience)

**Sub-theme 1.4:** Scarcity of mentors and professional support

The participants emphasised the need for accessible and supportive mentors to guide them through the research process:

‘The other thing there is lack of guidance from those that know to guide those that do not know, this is how this research will benefit us.’ (P1, Male, 12 years of experience)

‘Our supervisors and the people on top, they’re not giving us the support we had at school.’ (P2, Female, 2 years of experience)

‘It feels like once we leave school, we’re on our own. There’s no one to hold our hand or even check if we’re doing it right.’ (P4, Female, 1 year of experience)

**Sub-theme 1.5:** Limited personal benefit

Some participants expressed the view that engaging in research activities would not lead to tangible personal or professional benefits:

‘I do not see how participating in research will help me in my nursing career or improve my practice.’ (P5, Male, 5 years of experience)

‘Research feels like extra work that doesn’t directly impact my daily responsibilities or career growth.’ (P3, Male, 1 year of experience)

‘There are no incentives or rewards for nurses who get involved in research here, you just waste your time that you might use to rest.’ (P7, Male, 3 years of experience)

#### Theme 2: Organisational factors

This theme underlined the institutional elements that shape the research environment. Sub-themes included: (2.1) inadequate financial and material resources; (2.2) lack of dedicated research time; (2.3) absent research board committee and infrastructure; (2.4) absent leadership and management support; (2.5) sub-optimal research culture; and (2.6) lack of in-service training on the clinical research process.

**Sub-theme 2.1:** Inadequate financial and material resources

Participants reported a lack of funding, equipment and other material resources necessary to support research, as there is no dedicated budget for nursing research activities:

‘We don’t have the financial resources, or the equipment required to do meaningful research here. The hospital management is not willing to invest in this.’ (P1, Male, 12 years of experience)

‘Due to lack of finances due to the current economic situation. So, the ministry is also finding it hard to finance all the operational activities on the ground level or the district level and immediate level.’ (P1, Male, 12 years of experience)

‘The institution doesn’t provide the necessary infrastructure and materials to support nurses in conducting research.’ (P9, Male, 10 years of experience)

**Sub-theme 2.2:** Lack of dedicated research time

The participants expressed concerns about the scarcity of time to engage in research given their demanding clinical responsibilities:

‘So, for you to do research, you must have enough time. You must put in enough time.’ (P1, Male, 12 years of experience)

‘We are already stretched thin with our daily clinical duties, and there is no allotted time for us to work on research projects.’ (P12, Female, 11 years of experience)

‘With research, it will be hard for me because there is no time… and I cannot use my resting days to thin myself.’ (P15, Female, 15 years of experience)

**Sub-theme 2.3:** Absent research board committee and infrastructure

Participants highlighted the absence of a dedicated research board committee and the necessary infrastructure, such as research support staff and a centralised research office, to facilitate and coordinate nursing research efforts:

‘We do not have department that’s supposed to conduct research solely on its own.’ (P1, Male, 12 years of experience)

‘Not having a specific person employed for that specific task or not having a division about research that’s solely responsible for conducting operational research in the hospital.’ (P5, Male, 5 years of experience)

‘When I was an undergraduate student, I had a supervisor and here even if one wants to participate in research activities, one will not know how to move forward.’ (P12, Female, 11 years of experience)

**Sub-theme 2.4:** Absent leadership and management support

The participants reported a lack of encouragement and support from hospital leadership and management:

‘The hospital administration does not prioritise or promote nursing research. They are more focused on service delivery and patient care.’ (P13, Male, 14 years of experience)

‘We never hear anything from management about the importance of research. It’s just not on their agenda.’ (P10, Female, 14 years of experience)

‘Even when you want to do research, you face so many obstacles because management isn’t involved or interested.’ (P11, Male, 7 years of experience)

**Sub-theme 2.5:** Sub-optimal research culture

The participants expressed a perception of an organisational culture that does not sufficiently value or incentivise nursing research, which discourages their participation:

‘There’s no environment for research here. It’s all about getting the clinical work done, and research feels like an afterthought.’ (P8, Female, 17 years of experience)

‘The culture here is not so much about research; it’s more about clinical practice. Research is not seen as a priority or something that is actively encouraged or rewarded by the hospital administration.’ (P14, Female, 17 years of experience)

**Sub-theme 2.6:** Lack of in-service training on the clinical research process

The participants emphasised the need for comprehensive in-service training programmes to equip them with the necessary knowledge and skills to engage in clinical research effectively:

‘We also need to conduct maybe in-service training for the in-charges or for the ward supervisors regarding the importance of research and how we should use research to improve or to generate new knowledge.’ (P1, Male, 12 years of experience)

‘They should educate us on how research is done, how to do research, and how to go about if maybe we found a problem and want to research on it.’ (P2, Female, 2 years of experience)

‘Our supervisors and the people on top are not giving us the support we had from school, whereby they are supposed to guide us.’ (P2, Female, 2 years of experience)

#### Theme 3: Research factors

This theme captured the characteristics of the research process that affected the nurses’ participation and utilisation of clinical research. Sub-themes included: (3.1) complexity of the research process; (3.2) lack of communication and feedback; (3.3) insufficient guidance from research supervisors; and (3.4) absence of institutional mechanisms for research dissemination.

**Sub-theme 3.1:** Complexity of the research process

Participants perceived research as overly complex and time-consuming:

‘The research process is too complicated and overwhelming. It requires a lot of steps, paperwork, and bureaucracy that we just don’t have the time or energy to deal with.’ (P15, Female, 15 years of experience)

‘The steps involved in doing research, like getting ethical approval, collecting data, and analysing results, are very demanding and take a lot of effort that we simply don’t have as bedside nurses.’ (P8, Female, 17 years of experience)

‘The workload, there won’t be any enough time to do that research, as research will require more time.’ (P3, Male, 1 year of experience)

**Sub-theme 3.2:** Lack of communication and feedback

The participants highlighted the need for improved communication and feedback mechanisms to keep them informed and engaged:

‘You do your research, and at the end of the research… no action is to be taken into consideration.’ (P3, Male, 1 year of experience)

‘A lot of people have conducted research [*but it’s*] never used in the Ministry of Health or even at the hospitals.’ (P4, Female, 1 year of experience)

‘Okay, we’re doing all this research, we are researching nursing, we’re finding problems that need to be reassessed by the hospitals, or the Ministry of Health. But what are they, are they really using our research, things we are researching on?’ (P4, Female, 1 year of experience)

**Sub-theme 3.3:** Insufficient guidance from research supervisors

The participants felt that the lack of support from the mentors and colleagues about the nursing research process contributed to low participation and utilisation nursing research output as they could not conduct nursing research without aid:

‘The other thing is that maybe it’s just due to lack of guidance from those that knows to guide those that do not know that this is how we are supposed to do things, or this is how this research is going to benefit us.’ (P3, Male, 1 year of experience)

‘The support that we’re supposed to have here is the one like the one we had at school. Our supervisors and the people on top, they’re not giving us the support we had from school, whereby they are supposed to guide us.’ (P2, Female, 2 years of experience Female)

**Sub-theme 3.4:** Absence of institutional mechanisms for research dissemination

The participants emphasised the importance of establishing effective channels for disseminating research findings:

‘If someone conducts research [*there*] should be a platform where we can present our research findings to the wider nursing community, not just here in the hospital.’ (P9, Male, 10 years of experience)

‘There should be a mechanism where the research that’s been conducted in this hospital, they should be able to be presented or shared with other hospitals, so that we can learn from each other.’ (P10, Female, 14 years of experience)

‘but our research is never used in the Ministry of Health, or even at the hospitals, it is like we are just conducting research as a paper or as a qualification, but they’re not been used.’ (P4, Female, 1 year of experience)

## Discussion

This study explored the factors that affect nurses’ participation in, and utilisation of, clinical research within a regional hospital in Namibia. The findings highlighted a variety of factors, encompassing individual, organisational and research-related elements. Some concerns are the lack of formal knowledge about research methodologies, insufficient knowledge about the processes involved in conducting nursing research and limited capabilities. This aligns with the findings of Dagne and Ayalew (2020) and others, who discovered nurses’ poor attitudes towards research utilisation due to insufficient research skills (Hammad et al. 2020; Jabonete & Roxas 2022; Sowunmi et al. 2021). The findings show that nurses lack intrinsic motivation to conduct research and have a poor sense of self-efficacy in their ability to undertake research projects successfully. A similar study conducted in Nigeria reported that a lack of mentorships and research involvement had affected the motivation of nurses (Sowunmi et al. 2021). Similarly, Shatimwene and Ashipala (2023) reported in their study that academics were not motivated to do research for publication. They added that doing research with money from their own pockets was also demotivating and negatively affected research production. This study found that nurses only participate in research to fulfil post-graduate certificate requirements, which is as per Oluwatosin’s (2014) research in Nigeria.

Some participants noted that engaging in research would not lead to tangible personal or professional benefits, such as career advancement, recognition and incentives. A study conducted in Ghana reported that nurses’ participation in clinical research was publicised, which was seen as a benefit (Nkrumah et al. 2018). The nurses also face challenges due to inadequate research infrastructure, financing, resources and administrative support, which is consistent with a study that highlighted that these are significant obstacles to nurses’ involvement in research and its application (Nkrumah et al. 2018). This aligns with research indicating that healthcare organisations should prioritise providing resources to promote greater research participation among nurses, and effectively implement research findings in their working environments (Abuhammad et al. 2020; Alotaibi 2023; Kyei et al. 2023; Morrison et al. 2021). Further, there is a lack of support from hospital leadership and management, which is as per studies by Nashwan, Abujaber and Mansour (2016) and Alotaibi (2023). The study’s findings also highlighted the need for in-service training to prepare nurses for active participation in research, as well as the limited time for them to engage in research alongside their demanding clinical responsibilities. This is consistent with studies by Dagne and Ayalew (2020), Sarabia-Cobo et al. (2015), Shifaza et al. (2014), Abuhammad et al. (2020), Bal and Shiner (2019), Hagan and Walden (2016), Hammad et al. (2020), Musumadi et al. (2023) and Nor et al. (2021). In the study of Ashipala and Livingi (2021), participants raised the issue of not having enough time available because they had to find a balance between their academic and personal lives, which they found quite challenging.

Additionally, the absence of a dedicated research board committee and the necessary infrastructure affects research participation among nurses. Studies conducted in Tanzania similarly identified these as a barrier to research (Alotaibi 2023; Kengia et al. 2023). Implementing continuous professional development programmes such as workshops, mentorship programmes and opportunities for hands-on research experience could address this issue. The findings also suggest that the hospital administration could play a crucial role in fostering a research-conducive environment by establishing a dedicated research division, allocating financial resources and providing registered nurses with the necessary infrastructure and support. These findings are consistent with the existing literature, which has consistently identified organisational factors as crucial determinants of nurses’ research engagement factors (Bal & Sahiner 2019; Rojaye & Netangaheni 2023). By addressing these multifaceted factors, healthcare organisations can create a more supportive environment to encourage a research-oriented culture among nurses and enhance their involvement in clinical research.

Establishing strong local and international research collaborations is crucial for equipping frontline workers with the necessary skills and capabilities to conduct effective research (Mansour et al. 2021). Numerous studies have highlighted the manifold advantages of international cooperation in health research, including knowledge exchange, expertise sharing and increased funding (Franzen, Chandler & Lang 2017; Thomson et al. 2016). Additionally, collaborations facilitate joint engagement in problem identification, research proposal development, research execution, publication and establishing a community of practice. Furthermore, they foster continuous learning, generate knowledge to inform the design of interventions and policies and enhance services, infrastructure and financial resource availability (Matenga et al. 2019).

### Strengths and limitations

This study’s key strength is its detailed, qualitative investigation of the factors impacting registered nurses’ participation in, and utilisation of, clinical research within a resource-limited healthcare setting. The findings of the study can only be generalised to registered nurses working at Intermediate Hospital Rundu.

### Recommendations

Based on the findings of this study, the following recommendations are made about mechanisms for improving research output at the hospital.

#### Strengthening individual research capabilities

The participants pointed to knowledge gaps in understanding research, denoting the need to develop strategies aimed at building capacity for nursing staff at individual and hospital levels.

Continuous professional development programmes to enhance the participants’ research knowledge, skills and self-efficacy should be implemented.

#### Establishing dedicated research infrastructure and resources with a board committee

The results showed that although the nurses were aware of the need to conduct research, the lack of supporting structures limited the capacity and motivation of nursing staff to undertake research. There is a need for the hospital management to develop and establish a dedicated research infrastructure, including a centralised research office, a research board committee and the allocation of adequate resources (financial, material and human) to support nursing research activities.

#### Provision of financial support for research

The study findings revealed that a lack of funding was one of the major barriers to research output at the hospital. Hence, the hospital management should address the funding for conducting research. The participants suggested dedicating funding and resources to support nurses in conducting research, including equipment and materials.

#### Enhance research communication and dissemination

The study findings revealed that feedback to communicate and disseminate research results was limited. Efforts to enhance research study results feedback dissemination through an integrated approach by researchers and hospital management should be addressed as an attempt to motivate nursing staff to participate, utilise and conduct clinical research.

## Conclusion

The findings of this study highlight the variety of factors influencing registered nurses’ participation in, and utilisation of, clinical research in a regional public hospital in Namibia. These include insufficient research awareness and skills, motivation and self-efficacy, support, personal benefits, and financial and material resources. The findings highlight the need for a multifaceted approach to enhance nurses’ utilisation of clinical research within a regional public hospital in Namibia. Strengthening individual research capacities, establishing dedicated research infrastructure and resources, and enhancing the communication and dissemination of research findings will foster a research-oriented culture and facilitate the utilisation of clinical research. By addressing these factors, healthcare institutions can cultivate a research-oriented culture and empower nurses to actively engage in and apply clinical research. Through a comprehensive approach targeting research capacity building, dedicated infrastructure and resources, and effective communication strategies, the hospital can unlock the full potential of its nursing workforce as critical contributors to evidence-based practice and continuous quality improvement.
